# Treatment of onion skin waste using dielectric barrier discharge cold plasma processing

**DOI:** 10.1002/fsn3.4448

**Published:** 2024-09-03

**Authors:** Berna Senguler, Celale Kirkin, Hilal Donmez, Senanur Unal

**Affiliations:** ^1^ Department of Food Engineering, Faculty of Chemical and Metallurgical Engineering Istanbul Technical University Maslak, Istanbul Turkey; ^2^ Department of Gastronomy and Culinary Arts, Faculty of Applied Sciences Istanbul Gelisim University Avcilar, Istanbul Turkey

**Keywords:** antioxidant activity, cold plasma, color, microbial quality, onion skin, total phenolic content

## Abstract

Onion skin constitutes a major part of industrial food waste, and cold plasma technology can be employed in the treatment of onion skin. Onion skin waste was ground and exposed to dielectric barrier discharge cold plasma (DBDCP) at 40 kV for 10 or 20 min. Samples that were not DBDCP treated were used as the control. The changes in the color, microbial load, total phenolic content (TPC), and antioxidant activity of the onion skin waste upon treatment were evaluated. An increase in the b* and C* values of the onion skin powder (OSP) was obtained after the DBDCP treatment. The DBDCP process also decreased the total mesophilic aerobic bacteria and yeast–mold counts of the OSP by up to 0.92 and 0.97 log cfu/g (colony‐forming units per gram), respectively. In addition, the TPC and antioxidant activity, as determined by 2,2‐diphenyl‐2‐picrylhydrazyl (DPPH) scavenging activity and ferric‐reducing antioxidant power (FRAP) methods, were increased for up to 59% and 28%, respectively, depending on the treatment time. In conclusion, the findings of the study show that using DBDCP processing in the treatment of onion skin waste can reduce microbial count while enhancing TPC and antioxidant activity.

## Graphical abstract

Onion skin waste was ground and subjected to cold plasma technology. The changes in the microbial load, color, total phenolic content (TPC), and antioxidant activity were observed. Cold plasma decreased microbial count and improved total phenolic content and antioxidant activity.

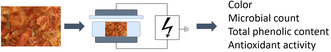

## 1 INTRODUCTION

Onion (*Allium cepa* L.) is an agricultural product that is widely cultivated and consumed in the world (Bello et al., 2013; Benítez et al., 2011). In addition, the generation of onion waste has been increasing with the increase in its production while threatening the environment (Benítez et al., 2011; Choi et al., 2015). The majority of onion waste is composed of onion skin, which has high antioxidant and antimicrobial properties and good fiber content (Benítez et al., 2011; Downes et al., 2009; Jaime et al., 2002; Škerget et al., 2009). Thus, onion skin can be employed as an ingredient in many culinary preparations (Gawlik‐Dziki et al., 2013; Kirkin et al., 2021; Kurt et al., 2019; Michalak‐Majewska, Teterycz, et al., 2020; Michalak‐Majewska, Złotek, et al., 2020; Sagar & Pareek, 2020, 2021). On the other hand, the microbial load of onion skin can be quite high and may also contain mycotoxins and pesticide residues (Katip, 2019; Zohri et al., 1992). Therefore, microbial safety must be controlled before the utilization of onion skin in food production.

Cold plasma processing has been one of the recent techniques that can be practiced in decontamination and pretreatment of food products (Elcik & Kirkin, 2024; Laroque et al., 2022; Niemira, 2012). Cold plasma can be created using various systems. In dielectric barrier discharge cold plasma (DBDCP) systems, the plasma is delivered between two electrodes when one or both of them are coupled with a material with dielectric properties (Kumar et al., 2023; Mandal et al., 2018; Niemira, 2012). It can enable microbial decontamination by inducing reactions between the plasma‐generated material and the membrane and damaging the membrane and cell components by ultraviolet (UV) irradiation produced during the treatment (Niemira, 2012). It has facilitated pathogenic and spoilage microorganism inactivation in several studies (Ahangari et al., 2021; Charoux et al., 2020; Chizoba Ekezie et al., 2017; Hemmati et al., 2021; Kashfi et al., 2020). Cold plasma technology can also be utilized in the oxidation and removal of gaseous pollutants (Veerapandian et al., 2019; Xie et al., 2024).

Furthermore, cold plasma can enhance bioactive compound extraction from plant material by disrupting the surface and improve the total phenolic content (TPC) and antioxidant activities (Bao et al., 2020; Farias et al., 2022; Heydari et al., 2023). Besides, cold plasma can negatively affect TPC and antioxidant properties depending on several parameters that are used in processing, such as treatment time, voltage, gas, etc. (Kumar et al., 2023). Thus, the conduction of further studies is needed to better comprehend the role of cold plasma process parameters in the TPC and antioxidant capacity of food products and to optimize the process.

No previous studies have examined the treatment of onion skin waste using cold plasma processing to the best of our knowledge. To evaluate the changes in the color values, microbial count, total phenolic content, and antioxidant activity of onion skin waste upon DBDCP was the objective of this study.

## 2 MATERIAL AND METHODS

### 2.1 Preparation and cold plasma treatment of the samples

The outer skins of yellow onion (*Allium cepa* L.) bulbs were collected as a waste after culinary class activity at Istanbul Gelisim University. Then, the onion skin waste was washed three times under tap water followed by 24‐h air‐drying on blotting paper to reach a moisture content of approximately 18% on a dry basis. Then, the onion skins were pulverized using a coffee grinder (Bosch, Türkiye) in order to have samples with uniform particle size (212–450 μm).

Three grams of onion skin powder (OSP) was placed in a glass petri dish (diameter: 10 cm, thickness: 1.5 mm). Then the petri dish was placed between two parallel electrodes (stainless steel, diameter: 12 cm, thickness: 4 mm) of the cold plasma reactor that was connected to a pulsed direct current (DC) power supply (Asentek, Ankara, Türkiye). The top electrode was connected to a glass plate (thickness: 2 mm), and the gap between the petri dish and the top glass barrier was 2 cm. The samples were treated with DBDCP at 40 kV (0.1 mA, 56 kHz) for 0 (control), 10, or 20 min. These parameters were selected according to the preliminary studies on different foods and surfaces. Each treatment was replicated three times.

### 2.2 Color measurement

The CIE color values (L*, a*, and b*) of the onion skin powder samples were determined using a color meter (CR‐400, Konica Minolta, Japan). A white plate was used to calibrate the device before the measurements. Three different measurements were averaged for each sample. Also, the Chroma (C*) and hue angle (h°) were calculated using the following formula:

$$C*=\sqrt{a{*}^{2}+b{*}^{2}}$$

$$h^{{}^{\circ}}=\arctan\;\left(\frac{b*}{a*}\right)$$

### 2.3 Microbial analysis

One gram of onion skin powder was diluted in buffered peptone water and spread on plate count agar (PCA) or dichloran rose bengal chloramphenicol agar (DRBC) for the total mesophilic aerobic bacteria (TMAB) count and total yeast–mold count, respectively. The plates for the TMAB and yeast–mold count were incubated for 48 h at 37°C and 3–5 days at 25°C, respectively.

### 2.4 Extraction of the samples

One gram of the OSP samples was added with 25 mL methanol (80%, v/v) and incubated at 40°C for 2 h in an ultrasonic bath (Isolab, Germany) and centrifuged at 4000 rpm (revolutions per minute) for 20 min (Universal 320R, Hettich, Germany). Then, the aliquots were separated and stored at −18°C.

### 2.5 Total phenolic content (TPC)

The TPC of the OSP samples was evaluated by the Folin–Ciocalteu method, as described before (Viuda‐Martos et al., 2010). Three hundred μL of OSP extract was added with 2.5 mL of Folin–Ciocalteu reagent (0.2 M) and 2 mL of sodium carbonate (Na_2_CO_3_) (7.5% in water, w/v) after diluting 8 times in methanol. Then, the mixtures were incubated at 50°C for 5 min, and the absorbance values were read at 760 nm using a spectrophotometer (T80 UV/VIS, PG Instruments, UK). Results were expressed as milligrams (mg) of gallic acid equivalents per gram (mg GAE/g) using a standard calibration curve.

### 2.6 Antioxidant activity

The 2,2‐diphenyl‐2‐picrylhydrazyl (DPPH) radical scavenging activity and ferric‐reducing antioxidant power (FRAP) assays were utilized to assess the antioxidant properties of the OSP samples (Viuda‐Martos et al., 2010). Fifty μL of extracts diluted in methanol was mixed with 2 mL of 6 × 10^−5^ M DPPH solution (in methanol) and incubated for 1 h at room temperature for the DPPH assay. Then, the absorbance of the mixtures was obtained at 517 nm. For the FRAP assay, one mL of OSP extract (diluted in methanol) was added with 2.5 mL of sodium phosphate (NaH_2_PO_4_) buffer (0.2 M, pH 6.6) and 2.5 mL of potassium ferricyanide (1%, w/v). A 2.5 mL of trichloroacetic acid (TCA) was added to the mixture after incubating for 20 min at 50°C. Then, a 2.5 mL of the mixture was mixed with 2.5 mL of distilled water and 0.5 mL of iron (III) chloride (1%, w/v), prior to incubation at room temperature for 30 min. Then, the absorbance was noted at 700 nm. The results obtained by both methods were expressed as milligrams (mg) of Trolox equivalents per gram (mg TE/g) using a standard calibration curve.

### 2.7 Statistical analysis

A full factorial design with three levels of DBDCP treatment and three replications was employed in the study. Data were assessed using statistical software (Minitab 16, Minitab, USA) by analysis of variance (ANOVA), Pearson's correlation, and Fisher's least significant difference (LSD) tests.

## 3 RESULTS AND DISCUSSION

### 3.1 Color

The changes in the color values of OSP, as affected by the DBDCP treatment, are given in Table 1. The C* values of the samples increased after DBDCP processing and with increasing treatment time (*p* < .05). Also, the b* value was higher after the DBDCP treatment for 20 min compared to other samples (*p* < .05), while no changes were observed in the other color values (L*, a*, and h°) of the samples (*p* > .05).

**TABLE 1:** Color values of the OSP samples as affected by the DBDCP treatment.

Treatment time (min)	L*	a*	b*	h°	C*
0	60.93 ± 0.32^a^	12.90 ± 0.18^a^	22.23 ± 0.08^b^	1.05 ± 0.01^a^	25.70 ± 0.02^c^
10	61.12 ± 0.66^a^	12.93 ± 0.13^a^	22.55 ± 0.13^b^	1.05 ± 0.01^a^	26.00 ± 0.06^b^
20	61.82 ± 0.42^a^	13.03 ± 0.06^a^	23.08 ± 0.14^a^	1.06 ± 0.00^a^	26.50 ± 0.09^a^

*Note*: Values are mean ± standard deviation of three replications and significantly different from those labeled with different superscript letters in the same column (*p* < .05).

In the present study, cold plasma‐induced changes were only observed on the b* and C* values. The increase in the b*‐ and C*‐values can be regarded as a desired effect of the cold plasma treatment for onion skins, as b* and C* values are related to yellowness and color saturation, respectively. It has been known that the major pigments in onion skins are quercetin and quercetin derivatives, which are known to have good antimicrobial and antioxidant properties (Choi et al., 2015; Lee, Patil, & Yoo, 2015; Ly et al., 2005; Osojnik Črnivec et al., 2021; Ramos et al., 2006; Yang et al., 2015). The b* and C* values positively correlated with the DPPH radical scavenging activity (*p* < .05). In addition, these values negatively correlated with the load of total yeast and mold (*p* < .05). Therefore, the increase in the antioxidant properties and the decrease in the fungal count can be related to the change in the color values. Moreover, color change occurred after cold plasma processing of several food products was reported in various studies. For instance, Rezaei et al. (2020) observed cold plasma‐induced significant color changes in hyssop leaves depending on the voltage and treatment time. Also, Darvish et al. (2022) stated that the color values (L*, a*, b*, C*, h°, and total color difference) of saffron increased after cold plasma processing. Moreover, cold plasma treatment of dried peppermint resulted in decreased L*, b*, and C* values and increased a* value and total color difference, as reported by Kashfi et al. (2020).

### 3.2 Microbial count

The differences in the TMAB and total yeast and mold counts on the onion skins after the treatments are shown in Table 2. A decrease (up to 0.92 log cfu/g) in the TMAB count was observed after DBDCP treatment compared to the control (*p* < .5). Also, the total yeast and mold count of the 20‐min DBDCP‐treated samples was significantly lower than that of the control and 10‐min treated samples (*p* < .05).

**TABLE 2:** Microbial count of the OSP samples as affected by the DBDCP treatment.

Treatment time (min)	Total viable (log cfu/g)	Yeast–mold (log cfu/g)
0	4.35 ± 0.05^a^	4.45 ± 0.18^a^
10	3.43 ± 0.13^b^	4.27 ± 0.01^a^
20	3.76 ± 0.04^b^	3.48 ± 0.18^b^

*Note*: Values are mean ± standard deviation of three replications and significantly different from those labeled with different superscript letters in the same column (p < .05).

The decreases in the TMAB and total yeast and mold counts were similar to the findings of Ahangari et al. (2021), and they stated that the total viable and mold count on dried walnut kernel was decreased by 1.09 and 0.89 log cfu/g, respectively, after cold plasma processing. Also, the total aerobic bacteria count on brown rice decreased with prolonged cold plasma exposure (Lee et al., 2016). The reduction in the fungal count on OSP after the DBDCP treatment was limited in the present study. This could be associated with the low water content of the samples, as the water in foods can be converted to free radicals (hydroxyl (OH), hydrogen ion (H^+^), and hydrogen peroxide (H_2_O_2_)) during plasma processing and improve the efficacy of the treatment (Bermudez‐Aguirre, 2020). The antimicrobial activity of cold plasma is lower for products with low water content (Bourke et al., 2017; Thirumdas et al., 2015). For instance, Lee, Kim, et al. (2015) observed that cold plasma‐induced reduction of pathogens on dried figs was lower than that on cabbage, and the efficacy of utilizing cold plasma was higher when the water activity was higher. It should also be added that the effect of cold plasma against foodborne microorganisms can be determined by many factors, such as the employed device, type of plasma, gas flow rate, gas composition, voltage, frequency, time, water content, surface, microorganism, etc. (Bourke et al., 2017; Mao et al., 2021; Misra et al., 2011; Niemira, 2012; Surowsky et al., 2015). The effectiveness of cold plasma increased with increasing exposure time, as claimed by several scholars (Darvish et al., 2022; Lee et al., 2016; Tappi et al., 2016). Thus, it could be possible to improve the reduction of microorganisms on OSP by changing the cold plasma processing parameters.

### 3.3 Total phenolic content

The DBDCP treatment significantly increased the TPC of onion skin waste (*p* < .05) as demonstrated in Table 3, while no difference was noted between the 10 and 20‐min treatments (*p* > .05).

**TABLE 3:** Total phenolic content of the OSP samples as affected by the DBDCP treatment.

Treatment time (min)	TPC (mg GAE/g)
0	5.47 ± 0.58^b^
10	12.02 ± 0.39^a^
20	10.12 ± 0.69^a^

*Note*: Values are mean ± standard deviation of three replications and significantly different from those labeled with different superscript letters in the same column (p < .05).

Cold plasma processing improved the TPC of onion skin waste in the present study. Several other studies also reported a similar finding. For instance, Bao et al. (2020) reported that 5‐ and 15‐min cold plasma treatments of grape pomace increased the TPC compared to control, while 10‐min treatment did not lead to a difference. In addition, an increase in the TPC of haskap after cold plasma treatment depending on the power was reported (Li et al., 2023). Moreover, Keshavarzi et al. (2020) reported that nitrogen (N_2_)‐cold plasma application at 15 W for 15 min augmented the TPC of green tea leaves, while air cold plasma (5–15 W, 5–15 min) caused the TPC to decrease. On the contrary, no changes in the TPC of black pepper grains were observed upon 3‐ and 5‐min cold plasma treatments at 15 and 30 kV (Charoux et al., 2020). The TPC of fresh and dried walnuts did not change after cold plasma exposure at 15 kV for up to 11 min (Sasireka et al., 2021). Furthermore, a previous study reported that the TPC value of ground dandelion root increased after 20‐min DBDCP treatment at 40 kV compared to control, while that of the unground roots decreased after 10‐min treatment (Elcik & Kirkin, 2024). In addition, Hemmati et al. (2021) claimed that increasing power and treatment time decreased the TPC of green tea powder. Also, a reduction in the TPC of red chili powder upon radio‐frequency cold plasma treatment was observed (Sasireka et al., 2021).

Cold plasma can disrupt the surface of plants, improve the extraction yield, and facilitate the release of bioactive compounds with the aid of plasma‐generated reactive species (Heydari et al., 2023). However, plasma‐generated reactive species can also reduce the amount of phenolics and antioxidant properties (Muhammad et al., 2018). These changes generally depended on the equipment setup, processing parameters, gasses, and food matrix (Muhammad et al., 2018). Thus, the conflicting findings reported previously could be related to the different cold plasma equipment, processing parameters employed, and the food matrix.

### 3.4 Antioxidant activity

The DBDCP‐induced differences in the antioxidant activity are exhibited in Table 4. The DPPH radical scavenging activity of the OSP samples was boosted by the DBDCP treatment for 20 min compared to the other samples (*p* < .05). The DPPH values of the 20‐min‐treated samples were greater than that of the control by 59%. The FRAP of the DBDCP‐treated samples was greater, by approximately 28% after the 10‐min treatment, than that of the control (*p* < .05).

**TABLE 4:** 2,2‐diphenyl‐2‐picrylhydrazyl (DPPH) and ferric‐reducing antioxidant power (FRAP) values of the OSP samples as affected by the DBDCP treatment.

Treatment time (min)	DPPH (mg TE/g)	FRAP (mg TE/g)
0	6.67 ± 0.45^b^	30.58 ± 3.53^b^
10	7.46 ± 0.61^b^	39.20 ± 2.04^a^
20	10.61 ± 0.45^a^	37.19 ± 1.86^a^

*Note*: Values are mean ± standard deviation of three replications and significantly different from those labeled with different superscript letters in the same column (p < .05).

Similar to the increase in the TPC, the DBDCP processing increased the antioxidant activity of the onion waste depending on the cold plasma exposure time and assessment method. Likewise, cold plasma process enhanced the DPPH scavenging activity of haskap powder, as reported by Li et al. (2023). Also, the DPPH scavenging activity of green coffee bean extract was improved by increasing treatment time up to 35 min, while a decrease was observed upon 60‐min treatment (Kungsuwan et al., 2023). The authors added that the FRAP of the green coffee bean extracts was not influenced by the 35‐min treatment. Similarly, the DPPH and FRAP values of peppermint extract increased upon cold plasma treatment at 50 W but decreased after 60‐W treatment (Kashfi et al., 2020). Furthermore, the DPPH and FRAP of ground dandelion root increased after 20‐min DBDCP treatment at 40 kV, but a decrease upon 10‐min treatment was noted in unground samples (Elcik & Kirkin, 2024).

## 4 CONCLUSIONS

The treatment of onion skin waste using DBDCP affected the color, microbial load, TPC, and antioxidant activity. The b* and C* values were increased after the treatment, and the highest values were obtained upon the treatment for 20 min. The DBDCP led to a reduction in the TMAB and yeast–mold counts. Furthermore, the DBDCP treatment improved the TPC, DPPH scavenging activity, and FRAP of the OSP. In conclusion, DBDCP processing can be used in onion skin waste treatment while improving the TPC and antioxidant properties and reducing the microbial load. However, it is worth adding that the effects of different cold plasma processing parameters (voltage, frequency, treatment time, feed gas, etc.) on the microbial inactivation and antioxidant and antimicrobial capacity of onion skin waste still need to be evaluated. The valorization of cold plasma‐treated onion skin waste as an ingredient in different food recipes will be assessed in further studies. It is also important that the mycotoxin levels and pesticide residues on onion skin waste are decreased before it is added to foods, and the influences of cold plasma on them still need to be determined.

## AUTHOR CONTRIBUTIONS

**Berna Senguler:** Formal analysis (equal); investigation (equal); methodology (equal); writing – original draft (equal). **Celale Kirkin:** Conceptualization (equal); methodology (equal); project administration (equal); supervision (equal). **Hilal Donmez:** Formal analysis (equal); investigation (equal). **Senanur Unal:** Formal analysis (equal); investigation (equal).

## FUNDING INFORMATION

This study was supported by the Scientific Research Projects Department of Istanbul Technical University (project number: MAB‐2021‐43242).

## CONFLICT OF INTEREST STATEMENT

The authors declare that there are no conflicts of interest.

## ETHICS STATEMENT

This study does not involve any human or animal testing.

## Data Availability

The data that support the findings of this study are available from the corresponding author upon reasonable request.
